# Should we start using colchicine for secondary prevention of acute and chronic coronary syndromes after 2024 European society of cardiology guidelines?

**DOI:** 10.2459/JCM.0000000000001688

**Published:** 2024-11-27

**Authors:** Massimo Imazio

**Affiliations:** a Department of Medicine (DMED), University of Udine; b Cardiothoracic Department, University Hospital Santa Maria della Misericordia, ASUFC, Udine, Italy

## Abstract

**Figure:**
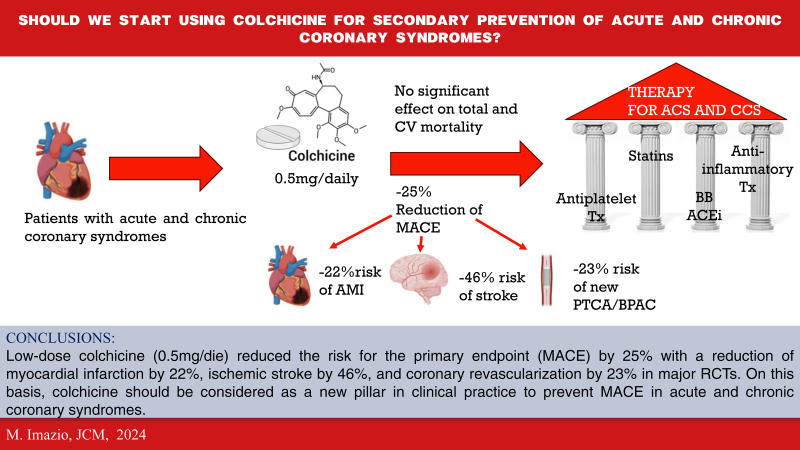

Colchicine is one of the oldest drugs used in medicine being mentioned for the first time in the Ebers papyrus (one of the oldest textbooks of medicine) in around 1550 BC. The drug is derived from the *Colchichum autumnale* plant.^1,2^ The name derives from the Colchys kingdom, an ancient region on the shores of the Black sea, where plants were widespread. The flowers of the plant are very similar to those of the saffron and accidental poisoning has been described due to improper use of the plant as saffron. This point underlines an important concept: colchicine has a narrow therapeutic window. Colchicine is usually studied as a mitosis inhibitor because of its blocking action on tubulin polymerization that interferes with the assembly of the microtubules. Probably for this reason, there is a common skepticism about the safety of the drug. However, it has been used for centuries to treat and prevent gouty attacks and it is well tolerated and efficacious to prevent the attacks of polyserositis (pericarditis and pleuritis) associated with a monogenic autoinflammatory disease such as the Familial Mediterranean Fever (FMF).^1,2^ It was starting from this efficacy that the drug was originally proposed to treat and prevent pericarditis at the end of the 80s by Antoni Bayes de Luna.^3^ Several subsequent spontaneous randomized controlled trials (RCTs) conducted in Italy have demonstrated the safety and efficacy of colchicine on top of standard therapy to prevent pericarditis by halving the recurrence rate, and speeding up the response to conventional anti-inflammatory therapy.^4–9^ On this basis, colchicine has been the first registered drug for pericarditis in Italy since April 2017.^4^ More recently, several spontaneous RCTs have demonstrated the efficacy and safety of low-dose colchicine (0.5 mg/day) to prevent major adverse cardiovascular events (MACE) in the setting of acute and chronic coronary syndromes.^10–12^ This viewpoint is aimed at reviewing the available evidence to support the routine use of colchicine for secondary prevention of coronary syndromes and guideline recommendations.

Since the CANTOS trial,^13^ which successfully demonstrated for the first time that anti-inflammatory therapy may prevent MACE in chronic ischemic heart disease, colchicine has been the second anti-inflammatory drug that has been efficacious for the same aim but with the advantages of having an oral route of administration and being very cheap. The preliminary observation that helped the development of trials with colchicine was the finding of decreased high-sensitivity levels of C-reactive protein after 30 days of treatment with colchicine in patients with stable coronary disease on optimal medical therapy.^14^

On this basis, it was possible to test the possible efficacy of low-dose colchicine (0.5 mg/daily) on clinical endpoints. In the first clinical trial (LoDoCo), open-label use of low-dose colchicine (0.5 mg/day) reduced MACE in patients with stable coronary artery disease [hazard ratio 0.33, 95% confidence interval (CI) 0.18–0.59], regardless of baseline CRP levels, on top of optimal medical therapy, including antiplatelet drugs and statins (Table 1).^10^

**Table 1:** Major clinical trials on the use of colchicine for secondary prevention of major cardiac adverse events in acute and chronic coronary syndromes

Study	Study design	Colchicine dose and duration	Clinical setting	Patient number	Main results
LoDoCo trial^10^ (2013)	Randomized trial (observer blinded)	Colchicine 0.5 mg daily for a median of 36 months plus statins and standard secondary prevention drugs	Stable coronary artery disease	532	Reduction of cardiovascular events (ACS, out-of-hospital cardiac arrest, noncardioembolic ischemic stroke): 5.3 vs. 16% (HR 0.33, 95% CI 0.18–0.59)
LoDoCo trial^11^ (2020)	Double-blind RCT	Colchicine 0.5 mg daily vs. placebo	Stable coronary artery disease	5522	Reduction of CV death, myocardial infarction, ischemic stroke, or ischemia-driven coronary revascularization: 6.8 vs. 9.6% (HR 0.69, 95% CI 0.57–0.83)
COLCOT trial^12^ (2019)	Double-blind RCT	Colchicine 0.5 mg daily for a median of 20 months	Recent myocardial infarction (<1 month)	4745	Reduction of cardiovascular events (composite of cardiovascular death, cardiac arrest, myocardial infarction, stroke or urgent hospitalizations for angina): 5.5 vs. 7.1% (HR 0.77, 95% CI 0.61–0.96)

ACS, acute coronary syndromes; CI, confidence interval; CV, cardiovascular; HR, hazard ratio; RCT, randomized controlled trial.

The LoDoCo trial made it possible to plan a double-blind larger RCT in the same population of chronic coronary syndromes: the LoDoCo2 trial, where a total of 5522 patients who were tolerant to an open-label treatment with colchicine 0.5 mg/daily were randomized to receive colchicine 0.5 mg/daily or placebo. In this trial, after a median follow-up of 29 months, colchicine significantly reduced the primary endpoint (cardiovascular death, spontaneous myocardial infarction, ischemic stroke, or ischemia-driven coronary revascularization) compared with the placebo (hazard ratio 0.69, 95% CI 0.57–0.83) without significant side effects.^11^

The subsequent major RCT evaluated the safety and efficacy of low-dose colchicine (0.5 mg/daily) in the setting of acute coronary syndromes for the first time. In the COLCOT patients with a recent (<1 month) myocardial infarction were randomized to colchicine or placebo and followed for up to 4 years. Also in this trial, colchicine reduced the incidence of the composite of cardiovascular death, cardiac arrest, myocardial infarction, stroke or urgent hospitalizations for angina (hazard ratio 0.77, 95% CI 0.61–0.96).^12^ A subsequent sub-study of COLCOT suggested that the efficacy improved with an earlier treatment when colchicine was initiated within 3 days of the onset of myocardial infarction (hazard ratio = 0.52, 95% CI 0.32–0.84).^15^ In the COLCOT trial, colchicine use was associated with a low but increased incidence of hospitalization for (nonfatal) pneumonia (0.9 vs. 0.4%, *P* = 0.03).^12^

Several systematic reviews have summarized the available evidence on colchicine for acute and chronic coronary syndromes. A meta-analysis pooling data from all of the major trials on colchicine in patients with acute and chronic coronary syndromes, including the results of the major five trials, included 11 816 patients and evaluated as the primary efficacy endpoint the composite of myocardial infarction, stroke, or cardiovascular death.^16^ In this systematic review, colchicine reduced the risk for the primary endpoint (MACE) by 25% (RR 0.75, 95% CI 0.61–0.92) with a reduction of myocardial infarction by 22%, ischemic stroke by 46%, and coronary revascularization by 23%. There was no difference in all-cause death (RR 1.08, 95% CI 0.71–1.62) with a trend for a lower incidence of cardiovascular death (RR 0.82, 95% CI 0.55–1.23), and a statistically not significant higher incidence of noncardiovascular death (RR 1.38, 95% CI 0.99–1.92),^16^ which gave rise to skepticism on the use of colchicine for secondary prevention in ischemic heart disease.

Probably on this basis, the 2021 European society of cardiology (ESC) guidelines on cardiovascular prevention introduced a shy IIa recommendation for the use of low-dose colchicine (0.5 mg once daily) in secondary prevention of cardiovascular disease (CVD), particularly if other risk factors are insufficiently controlled or if recurrent CVD events occur under optimal therapy, despite a level of evidence (LOE) A.^17^

On the contrary, based on current available evidence, in June 2023, the US Food and Drug Administration (FDA) approved the use of low-dose colchicine to reduce the risk of myocardial infarction, stroke, coronary revascularization, and cardiovascular death in adult patients with established atherosclerotic disease or with multiple risk factors for cardiovascular disease.^18^

More recently, the recent 2024 ESC guidelines on chronic coronary syndromes have upgraded the recommendation to class IIA LOE A without new significant publications (Table 2).^19^ This upgrade suggests a change of attitude that recognized the increasing importance of inflammation as a new target for cardiovascular medicine and anti-inflammatory drugs as a new possible pillar of therapy for coronary syndromes beyond antiplatelets, anticoagulants, beta blockers, ACE inhibitors, and statins.

**Table 2:** Food and Drug Administration registration and European guidelines recommendation on the use of colchicine for secondary prevention of major cardiac adverse events in patients with acute and chronic coronary syndromes

	Recommendation	Class and LOE
FDA registration^18^	Colchicine 0.5 mg/daily is approved for CV prevention to reduce the risk of myocardial infarction, stroke, coronary revascularization, and cardiovascular death in adult patients with established atherosclerotic cardiovascular disease (ASCVD) or with multiple risk factors for cardiovascular disease	n.a.
2021 ESC Guidelines for cardiovascular prevention^17^	Low-dose colchicine 0.5 mg p.o. daily should be considered for secondary prevention purposes, particularly among individuals with uncontrolled risk factors or recurrent events despite optimal medical therapy	IIb, LOE A
2024 ESC Guidelines on the management of chronic coronary syndromes^19^	In CCS patients with atherosclerotic CAD, low-dose colchicine (0.5 mg daily) should be considered to reduce myocardial infarction, stroke, and need for revascularization	IIa, LOE A

ESC, European Society of Cardiology; FDA, Food and Drug Administration; LOE, Level of Evidence; n.a., not applicable.

European medicines agency approval for secondary prevention of ischemic heart diseases is still pending in Europe.

There is a common unjustified skepticism on the safety of colchicine. These concerns probably are related to the well known antimitotic effect of colchicine, the evidence of an increased incidence of hospitalization for (nonfatal) pneumonia in the COLCOT trial, as well as evidence of a trend towards a higher incidence of noncardiovascular death reported in trials and systematic reviews. However, all these trends were statistically not significant.

A recent article reviewing safety data from the LoDoCo-2 trials has clearly demonstrated that the use of a low dose of colchicine is not associated with increased mortality.^20^ In the LoDoCo-2 trial, after a median follow-up 28.6 months, 133 out of 5522 participants (2.4%) died. Cardiovascular deaths were similar in patients with or without colchicine. Recorded noncardiovascular deaths included: cancer, end-stage pulmonary disease, infections, dementia, and multiple organ failure, all equally distributed in patients treated with or without colchicine. Multivariable analysis demonstrated that age of more than 65 years was the only independent baseline characteristic associated with noncardiovascular death (hazard ratio 3.65; 95% CI 2.06–6.47). Most deaths were related to noncardiovascular causes, underlying the importance of comorbidities in these patients.^20^

A recently published meta-analysis also pointed out clearly that the beneficial effects of the drugs can be achieved with low doses (0.5 mg/day). Low-dose colchicine significantly reduced MACE (RR 0.51; 95% CI 0.32–0.83), recurrent MI (RR 0.56; 95% CI 0.35–0.89), stroke (RR 0.48; 95% CI 0.23–1.00), and hospitalization (RR 0.44; 95% CI 0.22–0.85), whereas high and loading doses significantly increased gastrointestinal adverse events (RR 2.84; 95% CI 1.26–6.24) and discontinuation (RR 2.73; 95% CI 1.07–6.93).^21^

Although the therapeutic window of colchicine is narrow, low-dose colchicine (0.5 mg/daily) is well tolerated and efficacious for treatment, whereas loading doses and higher doses only increase the risk of side effects, intolerance, and drug withdrawal. It is noteworthy that data collected over the last 50 years strongly suggest that the biologic effects of long-term colchicine do not increase the risk of cancer, sepsis, cytopenia, or myotoxicity.^22^

In conclusion, there is well established evidence that inflammation is a therapeutic target for atherosclerotic cardiovascular diseases of similar, if not superior, importance to the reduction of low-density lipoprotein (LDL)-cholesterol, as demonstrated by Ridker *et al.*^23^ in a recently published collaborative international analysis. The study included 31 245 patients from the PROMINENT (*n* = 9988), REDUCE-IT (*n* = 8179), and STRENGTH (*n* = 13 078) trials. Hazard ratios for cardiovascular events and deaths were calculated across quartiles of high-sensitivity CRP and LDL-C in adjusted analyses. Residual inflammatory risk was significantly associated with MACE (highest high-sensitivity CRP quartile vs. lowest high-sensitivity CRP quartile, adjusted hazard ratio 1.31, 95% CI 1.20–1.43), cardiovascular mortality (hazard ratio 2.68, 95% CI 2.22–3.23), and all-cause mortality (hazard ratio 2.42, 95% CI 2.12–2.77). On the contrary, the relationship of residual cholesterol risk was neutral for MACE (highest LDLC quartile vs. lowest LDLC quartile, adjusted hazard ratio 1.07, 95% CI 0.98–1.17), and of low magnitude for cardiovascular death (hazard ratio 1.27, 95% CI 1.07–1.50) and all-cause death (hazard ratio 1.16, 95% CI 1.03–1.32).^23^

Nowadays, we already have a possible efficacious anti-inflammatory drug to be used in clinical practice according to the results of several studies, which have clearly demonstrated the safety and efficacy of low-dose colchicine for the secondary prevention of MACE in acute and chronic coronary syndromes.^10–12,24,25^ The FDA has already approved this indication based on currently available evidence that was produced by nonsponsored, spontaneous, investigator-initiated trials. Last but not least, colchicine is a cheap oral drug that could reduce MACE by 25–30% as well as statins, a class of drugs that nobody would deny to these patients. On this basis, we should now start prescribing low-dose colchicine to reduce MACE in our patients regardless of C-reactive protein levels,^10–12^ although hs-CRP greater than 2 mg/l could be considered as a marker of low-degree systemic inflammation and residual inflammatory risk in the single patient for individual risk assessment.^23–25^

## Conflicts of interest

There are no conflicts of interest.
